# Humans and Dolphins: Decline and Fall of Adult Neurogenesis

**DOI:** 10.3389/fnins.2018.00497

**Published:** 2018-07-20

**Authors:** Roberta Parolisi, Bruno Cozzi, Luca Bonfanti

**Affiliations:** ^1^NICO – Neuroscience Institute Cavalieri Ottolenghi, Turin, Italy; ^2^Department of Comparative Biomedicine and Food Science, University of Padua, Padua, Italy; ^3^Department of Veterinary Sciences, University of Turin, Turin, Italy

**Keywords:** adult neurogenesis, brain plasticity and aging, comparative anatomy, doublecortin, immature neurons

## Abstract

Pre-clinical research is carried out on animal models, mostly laboratory rodents, with the ultimate aim of translating the acquired knowledge to humans. In the last decades, adult neurogenesis (AN) has been intensively studied since it is viewed as a tool for fostering brain plasticity, possibly repair. Yet, occurrence, location, and rate of AN vary among mammals: the capability for constitutive neuronal production is substantially reduced when comparing small-brained, short living (laboratory rodents) and large-brained, long-living species (humans, dolphins). Several difficulties concerning scarce availability of fresh tissues, technical limits and ethical concerns did contribute in delaying and diverting the achievement of the picture of neurogenic plasticity in large-brained mammals. Some reports appeared in the last few years, starting to shed more light on this issue. Despite technical limits, data from recent studies mostly converge to indicate that neurogenesis is vestigial, or possibly absent, in regions of the adult human brain where in rodents neuronal addition continues into adult life. Analyses carried out in dolphins, mammals devoid of olfaction, but descendant of ancestors provided with olfaction, has shown disappearance of neurogenesis in both neonatal and adult individuals. Heterogeneity in mammalian structural plasticity remains largely underestimated by scientists focusing their research in rodents. Comparative studies are the key to understand the function of AN and the possible translational significance of neuronal replacement in humans. Here, we summarize comparative studies on AN and discuss the evolutionary implications of variations on the recruitment of new neurons in different regions and different species.

## Introduction

**FIGURE 1:**
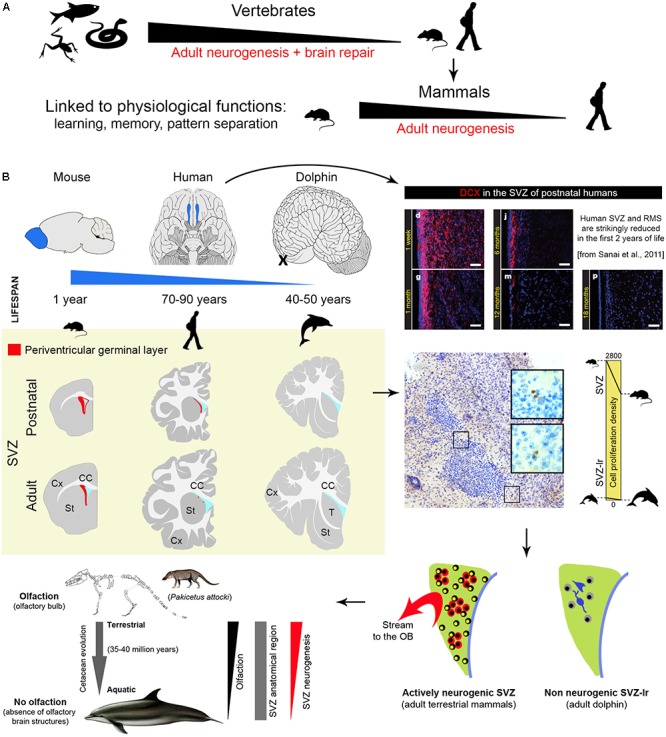
Comparison of central nervous system plasticity and adult neurogenesis in the animal world. **(A)** Strong reduction of brain neurogenic and reparative capacity occurs in vertebrates from non-mammals to mammals. **(B)** Among mammals, striking reduction is detectable in SVZ neurogenesis (a process which in laboratory rodents provides new neurons for the olfactory bulb throughout life) of large brained, long living species with reduced (humans) and absent (dolphins) olfactory brain structures. Images adapted from Sanai et al. (2011) and Parolisi et al. (2017) (reproduced with permission of Nature Publishing Group and Springer). *Yellow panel*: schematic representation of the reduction of SVZ neurogenesis in different animal species and at different ages (continuous red line, SGZ neurogenic niche; red dots, occurrence of scattered newly born/immature neurons).

After long debate since its first demonstration (Altman and Das, 1965), adult neurogenesis (AN) became accepted in birds in the 1980s by the direct illustration of long-range neuronal migration and the demonstration that the new cells had physiological properties of functional neurons (Paton and Nottebohm, 1984). Ten years later, the use of genetic tools to label the newborn neurons was functional to demonstrate the long migration and integration of new neurons in the mouse brain (Lois and Alvarez-Buylla, 1994; Suhonen et al., 1996). Laboratory rodents are considered by the vast majority of scientists the best (maybe the “only”) animal model for biomedical research and for translational science in humans. Comparative studies in other mammals are still considered as either oddities or scarcely useful duplicates. Mice and humans share striking biological similarities, but important differences and biases also emerge when complex biological processes are concerned (Bolker, 2017). Brain structural plasticity and its adaptation to different environment and differences in animal behavior are a typical example (Lipp and Bonfanti, 2016; Faykoo-Martinez et al., 2017). If basic neuronal stem cell biology can be similar in all mammals, the behavior of their differentiated neuronal progeny can substantially vary in brains whose neuroanatomies and development/postnatal growth also differ (Workman et al., 2013). It is well known that AN is highly reduced as to its rate, anatomical extension, reparative capacity when comparing non-mammalian vertebrates with mammals (Grandel et al., 2006; Bonfanti, 2011; Kempermann, 2016; **Figure 1A**). Mammals are often considered homogeneous in their capability to undergo structural plasticity, nevertheless, the occurrence, location and rate of neurogenesis substantially differ when comparing laboratory rodents with large-brained, long-living species (Lipp and Bonfanti, 2016; Paredes et al., 2016; Parolisi et al., 2017). This fact is still underestimated by many scientists working in the field. This mini-review is intended to draw attention to evolutionary issues linked to mammalian AN, in the light of recent studies carried out on humans and dolphins.

## Adult Neurogenesis in Mice and Humans: The Numbers

The two main neurogenic sites, the subventricular zone (SVZ) of the lateral ventricles and the subgranular zone (SGZ) in the hippocampal dentate gyrus (DG), are less active in adult humans than in adult rodents. Differences concerning the SVZ are quite striking: changes occurring in early postnatal human infants lead to the disappearance of the rostral migratory stream (RMS) around 18 months of age (Sanai et al., 2011). Then, only rare migrating neurons are observed in the SVZ and it remains unclear if these few cells can make the very long journey from the ventricle to their final destination in the olfactory bulb (OB; Sanai et al., 2011; Wang et al., 2011; Conover and Todd, 2017). The picture appears quite different in laboratory rodents, in which the SVZ is still active in adults and retains dividing stem cell populations throughout life. In mice, it provides continuous delivery of new neurons into the OB through chain migration in the RMS (estimated in ∼10,000 cells/day out of ∼180,000 newly generated cells in the whole SVZ region in a 3-month-old mice; Lois and Alvarez-Buylla, 1994; Ponti et al., 2013; Bordiuk et al., 2014). Nevertheless, stem cell activity/neurogenesis levels are quite reduced with age also in these rodents (Shook et al., 2012; Obernier et al., 2018).

**FIGURE 2:**
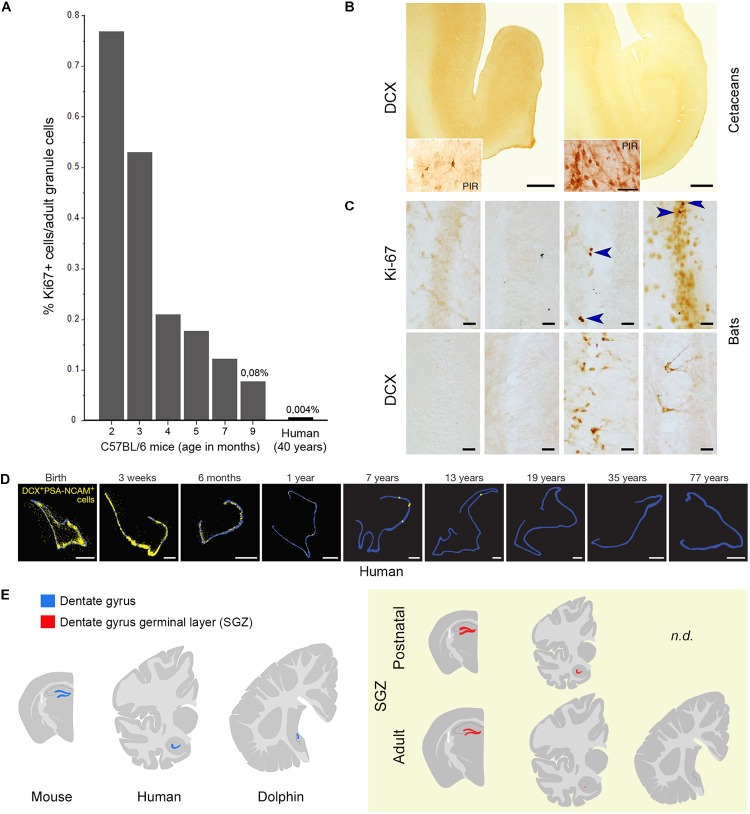
Substantial reduction or absence of neurogenic activity in the hippocampus of mice **(A)**, humans **(A,D)**, dolphins **(B)**, and bats **(C)**. **(A)** Percentage of Ki67+ cells as related to the total granule cell number (roughly corresponding to daily turnover) in C57BL/6 mice at different age levels (as reported in Ben Abdallah et al., 2010) and estimated percentage in human hippocampi as indicated by Spalding et al. (2013) and Bergmann et al. (2015); reproduced from Lipp and Bonfanti (2016), with permission of S. Karger AG, Basel. **(B)** No doublecortin (DCX) is detectable in the hippocampi of cetaceans (harbor porpoise, *Phocoena phocoena*, left; minke whale, *Balaenoptera acutorostrata*, right; adapted from Patzke et al., 2015 with permission from Springer); PIR, piriform cortex used as a positive control. **(C)** Absent or very low occurrence of cell proliferation (Ki-67 antigen) and immature neurons (DCX) in the hippocampus of bats; from left to right: *Phyllostomus discolor, Hipposideros cyclops, Hipposideros caffer, Mops condylurus*. Adapted from Amrein et al. (2007). Arrowheads: Ki-67+ nuclei. **(D)** The number of DCX+/PSA-NCAM+ cells (yellow) declines in the human dentate gyrus (blue outline) from infancy into childhood (reproduced from Sorrells et al., 2018 with permission of Nature Publishing Group). **(E)** *Yellow panel*: schematic representation of the reduction of dentate gyrus neurogenesis in different animal species and at different ages (continuous red line, SGZ neurogenic niche; red dots, occurrence of scattered newly born/immature neurons).

Adult hippocampal neurogenesis has been suggested to be retained into adulthood in different mammalian species (Amrein, 2015), including humans (Kempermann, 2016). Yet, a substantial reduction in the rate of neurogenesis occurs from young to adult age also in the DG of all species, including mice (in C57BL/6 mice, the reduction in proliferation is 10-fold, from 0.76 – percentage of Ki-67+ cells/granule cells – at 2 months to 0.08 at 9 months of age; Ben Abdallah et al., 2010). What about humans? A highly cited study published in cell and carried out using a technique based on incorporation of radioactive ^14^C left from nuclear explosions in the 1950s (Spalding et al., 2013) showed that the early decay of hippocampal neurogenesis in humans is less severe, claiming that its rate at 40 years of age is comparable with the C57BL/6 laboratory mouse at 9–12 months. In fact, the authors infer such “comparable” daily turnover of new granule cells (also reported in Bergmann et al., 2015) from a calculation based on a typo error in the Ben Abdallah et al.’s (2010) paper: the number expressing the negative exponential curve (changes in proliferating cells in the SGZ) is reported as *Y* = 1051, yet it was in reality *Y* = 10051. The mistake is easily identifiable by checking the raw data of the histograms reporting the number of Ki-67+ cells. The true relation (humans: 700 new cells out of 20 million granule cells = 0.0035%; mice at 9 months: 416 out of 0.5 million granule cells = 0.083%) is indicated in **Figure 2A**. Thus, men and mice appear to differ highly in hippocampal neurogenesis occurring at adult ages, the turnover rate in older humans being 10–20 times lower than in mice (see **Figure 2A** and Lipp and Bonfanti, 2016, for more detail). A similar rate has been found in the hippocampus of non-human primates, i.e., the adult macaque monkeys (Kornack and Rakic, 1999). Due to the technical limitations of using postmortem human brain samples and to a lack of robust, histological/immunocytochemical data, direct evaluation of human hippocampal neurogenesis remained open for several years. Then, three reports appeared in 2018. A detailed study carried out on postmortem and intraoperative samples of the human hippocampus showed that proliferating progenitors and young neurons in the DG sharply decline in the first year of life and only a few isolated young neurons can be detected by 7–13 years of age (Sorrells et al., 2018; **Figure 2D**). Very similar data emerge from another detailed report performed in the human hippocampus from early gestation to aging adults (Cipriani et al., 2018). These studies come to the conclusion that if AN continues in the adult DG, this process must be extremely rare. Finally, in a study claiming maintenance of neurogenesis in adult human hippocampus (Boldrini et al., 2018), actually various molecular markers were found associated to different stages of immature neurons, which do not show the typical aspect of recently generated neuroblasts. All these studies employed a large battery of antibodies on a great number of human specimens, indicating that most antigens are detectable in postmortem tissue (Arellano et al., 2018). The Sorrells et al.’s (2018) work performed the more complete histologic analysis (whole sections of SGZ neurogenic site examined through ages). All papers substantially show a similar landscape, though the interpretation of data highly differ in the Boldrini report. Indeed, even in the absence of constitutive niches, genesis of isolated neurons cannot be excluded. Further studies involving post-surgical resection work and single cell RNAseq from human neurogenic zones may help to define their developmental potential over time.

The majority of mammals does show adult hippocampal neurogenesis to some extent, with exceptions in dolphins, humans and some bats (Amrein et al., 2007; Patzke et al., 2015; Sorrells et al., 2018; **Figures 2B,C,E**). Neurogenesis seems to be under selective pressure. Under an evolutionary profile, humans have it during the youngest ages that likely had the greatest phylogenetic importance in the past. Open questions about adult human neurogenesis include: (i) are low levels of neurogenesis functionally relevant? (ii) are there vestigial/quiescent remnants of stem cell niches and can these be reactivated in some way? Some authors, considering that the new neurons within the DG, even a low number, can be highly functional (at least in animal models), argue that “there has been evolution toward neurogenesis-based plasticity rather than away from it” (Kempermann, 2016). At present, no systematic, fully comparable studies are available on a wide range of mammalian species to support this view or evaluate the importance of conservation in AN. Really comparable studies would involve the count of dividing and DCX+ cells, in relation to the count of the different cell populations residing in the hippocampus of different species (primarily granule cells, whose number can undergo substantial individual variations: between 10 and 30 million in humans; Simic et al., 1997). An existent analysis, mostly based in rodent species (van Dijk et al., 2016) indicates that the rate of the neurogenesis varies widely, either due to differences in the rates of neuronal birth or to different rates in neurogenesis decline. A wider, systematic analysis involving different mammalian orders is lacking.

## Absence of Postnatal Neurogenesis in the Dolphin Brain

Dolphins are large-brained, highly gyrencephalic, long-living mammals endowed with high cognitive abilities and sophisticated navigation systems (Marriott et al., 2013). The adaptation to aquatic life promoted the evolution of features (echolocation, composite language, capacity to elaborate intricate social skills) related to their ecological niche and sometimes similar in complexity to humans (Cozzi et al., 2017; van Kann et al., 2017). Among several aspects worthy of a comparative study on neurogenic activity in dolphins, we focused on a unique trait: the absence of olfaction/olfactory brain structures (Oelschläger, 2008; Berta et al., 2014; Cozzi et al., 2017). This is not simply due to dolphins not needing olfaction because of their aquatic adaptation. Fish have olfaction and olfactory systems similar to that of other vertebrates, supporting behaviors crucial for survival (Kermen et al., 2013). Instead, dolphins have developed echolocation for navigation, foraging, and tracking of prey (Marriott et al., 2013), thus, unlike terrestrial mammals (and fish), toothed whales have completely lost olfaction (Oelschläger, 2008; Cozzi et al., 2017). Since the SVZ of the lateral ventricles provides neuronal progeny destined for the OB and linked to olfactory discrimination (Lledo and Valley, 2016; Zhuo et al., 2016), we investigated the periventricular region of neonatal and adult dolphins in search for neurogenic activity. The typical germinal layer described in neonatal mammals (Tramontin et al., 2003; Peretto et al., 2005) was absent at birth in dolphins (Parolisi et al., 2015), replaced by a vestigial remnant (SVZ-like region; SVZ-lr) hosting small spots of non-proliferating cells beneath the ventricular wall (Parolisi et al., 2017; **Figure 1B**). In the neonatal SVZ-lr, Ki-67 antigen localization revealed very low numbers (negligible density) of dividing cells: 34-fold lower than in the germinal layer of the cerebellar cortex of the same animals, 62-fold lower than in the SVZ of neonatal rodents, 47-fold lower than in adult rodents (**Figure 1B**). A similar, very small number of SVZ-lr Ki-67 labeled cells was found in adults (Parolisi et al., 2017). The SVZ-lr area was similar to that in mice, whose brain is 3000-fold smaller if the weight or volume are considered. In dolphins, soon after birth, it appears compartmentalized into cell clusters (a feature reminiscent of AN sites), intermingled with neurons that express mature neuronal markers. Hence, in the dolphin SVZ-lr an early exhaustion of cell division is followed by a local, unknown rate of neuronal maturation. The absence of clear signs of active neurogenesis in aquatic mammals devoid of working olfaction/OB is apparently in contrast with the existence of an SVZ-lr throughout their lifespan. The explanation might be found in their evolutionary history, that started as terrestrial Cetartiodactyls that returned to the sea 35–40 million years ago (Thewissen et al., 2001). The terrestrial ancestors of dolphins were wolf-sized mammals endowed with olfactory structures (*Pakicetus*; Kishida et al., 2015), that underwent a gradual transition from land to sea, losing along the way the capacity to perceive odors (Thewissen et al., 2001). Thus, the vestigial, short-lived SVZ in humans, along with the not working SVZ-lr in dolphins, strongly support the view that AN is maintained in evolution only depending on strict relationships with its functional need(s).

## Why is Adult Neurogenesis Highly Reduced or Absent in Some Mammals?

The most likely explanation for the general reduction of AN in humans with respect to rodents might be related to the reduced importance of specific brain functions linked to survival, somehow replaced by other (higher) cognitive functions. This potential explanation acquires relevance when olfaction/olfactory brain structures (SVZ neurogenesis) are concerned. Although olfaction in humans is considered more impactful than previously thought (in term of total amount of neurons; McGann, 2017), the relative size of the OB with respect to the whole brain volume (0.01% of the human brain compared to 2% of the mouse brain) and the importance of olfaction for survival are quite reduced when compared to rodents (see **Figure 1B**). For this reason, we recently expressly searched the periventricular region of dolphins for neurogenic processes. The persistence of a vestigial remnant (functionally inactive) of the SVZ neurogenic niche in dolphins strongly suggests that periventricular neurogenesis reduction/disappearance occurs in parallel with reduction/disappearance of olfactory brain structures across evolution (Parolisi et al., 2017). Previous observations failing to detect DCX+ cells in the hippocampus of cetaceans (Patzke et al., 2015) suggest that AN does not continue into adult life in toothed whales. Interestingly, another group of mammals showing absence, or very low levels, of hippocampal neurogenesis, the bats (especially Microchiroptera; Amrein et al., 2007; Amrein, 2015; Chawana et al., 2016), are also endowed with echolocation. Unlike dolphins, bats possess a well-developed SVZ neurogenic niche and RMS (olfaction is important in their life; Jones et al., 2013), thus confirming the mutual relationships between the occurrence/rate of AN, its function, and the ecological niches in which a particular species evolved (see Amrein, 2015). Anyway, the link between low neurogenesis – long lifespan – large brain is broken up by the microchiroptera and naked mole-rats, both long-lived species but with small brains, thus adding further levels of complexity.

How these data can be put together in the context of AN reduction? Several studies indicate that olfactory systems held a paramount importance in early mammalian evolution working as a reference system for spatial navigation for the location of food sources and mates (Rowe et al., 2011; Jacobs, 2012). These olfactory systems were mostly linked to paleocortical–hippocampal structures, subsequently replaced/integrated by the expansion of the isocortex as a “multimodal interface” for behavioral navigation based on vision and audition (Aboitiz and Montiel, 2015). Indeed, with respect to brain mass, the isocortex has “positive allometry”: larger mammalian brains become progressively more composed of cortex, ranging from under 20% in relative volume in small shrews and rodents to over 80% in humans (Hofman, 1989; Finlay and Darlington, 1995). From an evolutionary perspective, the major expansion of the isocortex possibly took place “when other senses (vision, audition) began to provide information to the hippocampus to generate multimodal, bidimensional orientation maps” (Aboitiz and Montiel, 2015) and/or to improve long-term memory (e.g., in primates; Reep et al., 2007). Cetaceans did not develop vision to the extent of primates, but essentially base their orientation and navigation on echolocation, a combination of sound emission and perception that requires no olfactory structures for the detection of faraway targets (Marriott et al., 2013).

In the complexity of mammalian plasticity, research focused on single animal species or directed only to highly standardized models, minimizes genetic and environmental variation and may result misleading in a translational perspective (Bolker, 2017). If AN is concerned, models based on laboratory rodents are too simplistic if we consider the different neuroanatomies and the species-specific adaptations of mammals (Lipp and Bonfanti, 2016; Faykoo-Martinez et al., 2017). For instance, in a recent report, no significant change in the number of newly born neurons was detectable in young sheep forced to exercise (Swanson et al., 2017), sharply in contrast with rodents (Vivar and van Praag, 2017). The baseline exercise for lambs only consists of brief episodes of exploratory play and feeding activities, in contrast with the long-sustained periods of exercise required by rodents for feeding and survival. This difference further highlights how AN modulation is linked to species-specific natural behaviors (function-based need), rather than to a stimulus *per se*. Indeed, laboratory mice are not even a model for their wild counterpart, since physical activity (or the lack of) does not alter neurogenesis in wild rodents (Amrein, 2015).

Interestingly, in humans, primates, dolphins or naked mole rat, newly born DCX+ neurons can persist in the neurogenic areas long after their proliferation in a sort of prolonged maturation that does not take place in short-living rodents (Kornack and Rakic, 1999; Knoth et al., 2010; Kohler et al., 2011; Brus et al., 2013; Penz et al., 2015; Parolisi et al., 2017). The maturation of neurons in long-living species might be delayed to compensate for the strong reduction in the number of new neurons. Interestingly, the recent studies in the adult human hippocampus essentially show the existence of various kinds/stages of immature neurons.

## Conclusions and Future Perspectives

Three features of AN are crucial when considering its translational value: (i) its substantial decrease in humans and other long-living, large-brained mammals; (ii) its decrease with the age of the individuals (in both SVZ and hippocampus); and (iii) a scarce propensity/efficacy for lesion-induced repair in mammals. These constraints seem to strongly depend on evolutionary pathways (Weil et al., 2008). Evolution drives the occurrence, rate and type of plasticity among mammals, and interspecies differences must be taken into account when translating results from mice to humans. In parallel, mechanistic studies in mice may still guide future induction efforts or transplantation in humans. Current efforts are aimed at identifying and fostering the endogenous/exogenous sources of stem cells. However, future angles should also contemplate the search for other forms of plasticity potentially adopted by different species in alternative or in addition to the genesis of new neurons. In long-living species, it is more common to find DCX+ neurons which maintain markers of immaturity for a long time. This suggests that other forms of plasticity might compensate the loss of continuous neurogenesis (apparently not compatible with the acquisition of higher cognitive functions). An example consists of the so called “immature neurons”: non-newly generated, DCX+ cells which are born prenatally but persist through time in an immature state in non-neurogenic regions (Gómez-Climent et al., 2008; functional hypothesis reviewed in Bonfanti and Nacher, 2012; König et al., 2016). We recently showed that immature neurons are by far more present in sheep than in rodents, thus supporting the existence of distinct, possibly alternative, forms of structural plasticity in some mammals (Piumatti et al., 2017; Palazzo et al., 2018).

## Author Contributions

LB wrote the article. BC and RP contributed to writing the article.

## Conflict of Interest Statement

The authors declare that the research was conducted in the absence of any commercial or financial relationships that could be construed as a potential conflict of interest.

## References

[B1] Aboitiz F. Montiel J. F. (2015). Olfaction, navigation, and the origin of isocortex. *Front. Neurosci.* 9:402. 10.3389/fnins.2015.00402 26578863 PMC4621927

[B2] Altman J. Das G. D. (1965). Post-natal origin of microneurones in the rat brain. *Nature* 207 953–956. 10.1038/207953a0 5886931

[B3] Amrein I. (2015). Adult hippocampal neurogenesis in natural populations of mammals. *Cold Spring Harb. Perspect. Biol.* 7:a021295. 10.1101/cshperspect.a021295 25934014 PMC4448614

[B4] Amrein I. Dechmann D. K. Winter Y. Lipp H. P. (2007). Absent or low rate of adult neurogenesis in the hippocampus of bats (Chiroptera). *PLoS One* 2:e455. 10.1371/journal.pone.0000455 17520014 PMC1866182

[B5] Arellano J. I. Harding B. Thomas J. L. (2018). Adult human hippocampus: no new neurons in sight. *Cereb. Cortex* 28 2479–2481. 10.1093/cercor/bhy106 29746611

[B6] Ben Abdallah N. M. Slomianka L. Vyssotski A. L. Lipp H. P. (2010). Early age-related changes in adult hippocampal neurogenesis in C57 mice. *Neurobiol. Aging* 31 151–161. 10.1016/j.neurobiolaging.2008.03.002 18455269

[B7] Bergmann O. Spalding K. L. Frisen J. (2015). Adult neurogenesis in humans. *Cold Spring Harb. Perspect. Biol.* 7:a018994. 10.1101/cshperspect.a018994 26134318 PMC4484963

[B8] Berta A. Ekdale E. G. Cranford T. W. (2014). Review of the cetacean nose: form, function, and evolution. *Anat. Rec.* 297 2205–2215. 10.1002/ar.23034 25312374

[B9] Boldrini M. Fulmore C. A. Tartt A. N. Simeon L. R. Pavlova I. Poposka V. (2018). Human hippocampal neurogenesis persists throughout aging. *Cell Stem Cell* 22:589-599.e5. 10.1016/j.stem.2018.03.015 29625071 PMC5957089

[B10] Bolker J. A. (2017). Animal models in translational research: Rosetta stone or stumbling block? *Bioessays* 39:1700089. 10.1002/bies.201700089 29052843

[B11] Bonfanti L. (2011). From hydra regeneration to human brain structural plasticity: a long trip through narrowing roads. *ScientificWorldJournal* 11 1270–1299. 10.1100/tsw.2011.113 21666994 PMC5720118

[B12] Bonfanti L. Nacher J. (2012). New scenarios for neuronal structural plasticity in non-neurogenic brain parenchyma: the case of cortical layer II immature neurons. *Prog. Neurobiol.* 98 1–15. 10.1016/j.pneurobio.2012.05.002 22609484

[B13] Bordiuk O. L. Smith K. Morin P. J. Semenov M. V. (2014). Cell proliferation and neurogenesis in adult mouse brain. *PLoS One* 9:e111453. 10.1371/journal.pone.0111453 25375658 PMC4222938

[B14] Brus M. Meurisse M. Gheusi G. Keller M. Lledo P. M. Levy F. (2013). Dynamics of olfactory and hippocampal neurogenesis in adult sheep. *J. Comp. Neurol.* 521 169–188. 10.1002/cne.23169 22700217

[B15] Chawana R. Patzke N. Alagaili A. N. Bennett N. C. Mohammed O. B. Kaswera-Kyamakya C. (2016). The distribution of Ki-67 and doublecortin immunopositive cells in the brains of three Microchiropteran. species, Hipposideros fuliginosus, Triaenops persicus, and Asellia tridens. *Anat. Rec.* 299 1548–1560. 10.1002/ar.23460 27532288

[B16] Cipriani S. Ferrer A. Arinica I. Kovacs G. Verney C. Nardelli J. (2018). Hippocampal radial glial subtypes and their neurogenic potential in human fetuses and healthy and Alzheimer disease adults. *Cereb. Cortex* 28 2458–2478. 10.1093/cercor/bhy096 29722804

[B17] Conover J. C. Todd K. L. (2017). Development and aging of a brain neural stem cell niche. *Exp. Gerontol.* 94 9–13. 10.1016/j.exger.2016.11.007 27867091 PMC5435549

[B18] Cozzi B. Huggenberger S. Oelschläger H. H. A. (eds). (2017). “Chapter 6: brain, spinal cord, and cranial nerves,” in *The Anatomy of Dolphins: Insights into Body Structure and Function* (London: Academic Press), 191–285.

[B19] Faykoo-Martinez M. Toor I. Holmes M. M. (2017). Solving the neurogenesis puzzle: looking for pieces outside the traditional box. *Front. Neurosci.* 11:505. 10.3389/fnins.2017.00505 28943837 PMC5596094

[B20] Finlay B. L. Darlington R. B. (1995). Linked regularities in the development and evolution of mammalian brains. *Science* 268 1578–1584. 10.1126/science.7777856 7777856

[B21] Gómez-Climent M. A. Castillo-Gómez E. Varea E. Guirado R. Blasco-Ibáñez J. M. Crespo C. (2008). A population of prenatally generated cells in the rat paleocortex maintains an immature neuronal phenotype into adulthood. *Cereb. Cortex* 18 2229–2240. 10.1093/cercor/bhm255 18245040

[B22] Grandel H. Kaslin J. Ganz J. Wenzel I. Brand M. (2006). Neural stem cells and neurogenesis in the adult zebrafish brain: origin, proliferation dynamics, migration and cell fate. *Dev. Biol.* 295 263–277. 10.1016/j.ydbio.2006.03.040 16682018

[B23] Hofman M. A. (1989). On the evolution and geometry of the brain in mammals. *Prog. Neurobiol.* 32 137–158. 10.1016/0301-0082(89)90013-0 2645619

[B24] Jacobs L. F. (2012). From chemotaxis to the cognitive map: the function of olfaction. *Proc. Natl. Acad. Sci. U.S.A.* 109 10693–10700. 10.1073/pnas.1201880109 22723365 PMC3386877

[B25] Jones G. Teeling E. C. Rossiter S. J. (2013). From the ultrasonic to the infrared: molecular evolution and the sensory biology of bats. *Front. Physiol.* 4:117. 10.3389/fphys.2013.00117 23755015 PMC3667242

[B26] Kempermann G. (2016). Adult neurogenesis: an evolutionary perspective. *Cold Spring Harb. Perspect. Biol.* 8:a018986. 10.1101/cshperspect.a018986 26684183 PMC4743076

[B27] Kermen F. Franco L. M. Wyatt C. Yaksi E. (2013). Neural circuits mediating olfactory-driven behavior in fish. *Front. Neural Circuits* 7:62. 10.3389/fncir.2013.00062 23596397 PMC3622886

[B28] Kishida T. Thewissen J. G. M. Hayakawa T. Imai H. Agata K. (2015). Aquatic adaptation and the evolution of smell and taste in whales. *Zoological Lett.* 1:9 10.1186/s40851-014-0002 PMC4604112 26605054

[B29] Knoth R. Singec I. Ditter M. Pantazis G. Capetian P. Meyer R. P. (2010). Murine features of neurogenesis in the human hippocampus across the lifespan from 0 to 100 years. *PLoS One* 5:e8809. 10.1371/journal.pone.0008809 20126454 PMC2813284

[B30] Kohler S. J. Williams N. I. Stanton G. B. Camerond J. L. Greenough W. T. (2011). Maturation time of new granule cells in the dentate gyrus of adult macaque monkeys exceeds six months. *Proc. Natl. Acad. Sci. U.S.A.* 108 10326–10331. 10.1073/pnas.1017099108 21646517 PMC3121825

[B31] König R. Benedetti B. Rotheneichner P. O’Sullivan A. Kreutzer C. Belles M. (2016). Distribution and fate of DCX/PSA-NCAM expressing cells in the adult mammalian cortex: a local reservoir for adult cortical neuroplasticity? *Front. Biol.* 11 193–213. 10.1007/s11515-016-1403-5

[B32] Kornack D. R. Rakic P. (1999). Continuation of neurogenesis in the hippocampus of the adult macaque monkey. *Proc. Natl. Acad. Sci. U.S.A.* 96 5768–5773. 10.1073/pnas.96.10.5768 10318959 PMC21935

[B33] Lipp H. P. Bonfanti L. (2016). Adult neurogenesis in mammals: variations and confusions. *Brain Behav. Evol.* 87 205–221. 10.1159/000446905 27560356

[B34] Lledo P. M. Valley M. (2016). Adult olfactory bulb neurogenesis. *Cold Spring Harb. Perspect. Biol.* 8:a018945. 10.1101/cshperspect.a018945 27235474 PMC4968158

[B35] Lois C. Alvarez-Buylla A. (1994). Long-distance neuronal migration in the adult mammalian brain. *Science* 264 1145–1148. 10.1126/science.8178174 8178174

[B36] Marriott S. Cowan E. Cohen J. Hallock R. M. (2013). Somatosensation, echolocation, and underwater sniffing: adaptations allow mammals without traditional olfactory capabilities to forage for food underwater. *Zoolog. Sci.* 30 69–75. 10.2108/zsj.30.69 23387839

[B37] McGann J. P. (2017). Poor human olfaction is a 19th-century myth. *Science* 356:eaam7263. 10.1126/science.aam7263 28495701 PMC5512720

[B38] Obernier K. Cebrian-Silla A. Thomson M. Parraguez J. I. Anderson R. Guinto C. (2018). Adult neurogenesis is sustained by symmetric self-renewal and differentiation. *Cell Stem Cell* 22 221–234.e8. 10.1016/j.stem.2018.01.003 29395056 PMC5802882

[B39] Oelschläger H. H. A. (2008). The dolphin brain-a challenge for synthetic neurobiology. *Brain Res. Bull.* 75 450–459. 10.1016/j.brainresbull.2007.10.051 18331914

[B40] Palazzo O. La Rosa C. Piumatti M. Bonfanti L. (2018). Do large brains of long-living mammals prefer non-newly generated, immature neurons? *Neural Regen. Res.* 13 633–634. 10.4103/1673-5374.230282 29722307 PMC5950665

[B41] Paredes M. F. Sorrells S. F. Garcia-Verdugo J. M. Alvarez-Buylla A. (2016). Brain size and limits to adult neurogenesis. *J. Comp. Neurol.* 524 646–664. 10.1002/cne.23896 26417888 PMC5047485

[B42] Parolisi R. Cozzi B. Bonfanti L. (2017). Non-neurogenic SVZ-like niche in dolphins, mammals devoid of olfaction. *Brain Struct. Funct.* 222 2625–2639. 10.1007/s00429-016-1361-3 28238073

[B43] Parolisi R. Peruffo A. Messina S. Panin M. Montelli S. Giurisato M. (2015). Forebrain neuroanatomy of the neonatal and juvenile dolphin (*T. truncatus* and *S. coeruloalba)*. *Front. Neuroanat.* 9:140. 10.3389/fnana.2015.00140 26594155 PMC4635206

[B44] Paton J. A. Nottebohm F. (1984). Neurons generated in adult brain are recruited into functional circuits. *Science* 225 1046–1048. 10.1126/science.6474166 6474166

[B45] Patzke N. Spocter M. A. Karlsson K. Æ. Bertelsen M. F. Haagensen M. Chawana R. (2015). In contrast to many other mammals, cetaceans have relatively small hippocampi that appear to lack adult neurogenesis. *Brain Struct. Funct.* 220 361–383. 10.1007/s00429-013-0660-1 24178679 PMC8734557

[B46] Penz O. K. Fuzik J. Kurek A. B. Romanov R. Larson J. Park T. J. (2015). Protracted brain development in a rodent model of extreme longevity. *Sci. Rep.* 5:11592. 10.1038/srep11592 26118676 PMC4484490

[B47] Peretto P. Giachino C. Aimar P. Fasolo A. Bonfanti L. (2005). Chain formation and glial tube assembly in the shift from neonatal to adult subventricular zone of the rodent forebrain. *J. Comp. Neurol.* 487 407–427. 10.1002/cne.20576 15906315

[B48] Piumatti M. Palazzo O. La Rosa C. Crociara P. Parolisi R. Luzzati F. (2017). Non-newly generated, “immature” neurons in the sheep brain are not restricted to cerebral cortex. *J. Neurosci.* 38 826–842. 10.1523/JNEUROSCI.1781-17.2017 29217680 PMC6596233

[B49] Ponti G. Obernier K. Guinto C. Jose L. Bonfanti L. Alvarez-Buylla A. (2013). Cell cycle and lineage progression of neural progenitors in the ventricular-subventricular zones of adult mice. *Proc. Natl. Acad. Sci. U.S.A.* 110 E1045–E1054. 10.1073/pnas.1219563110 23431204 PMC3600494

[B50] Reep R. L. Finlay B. L. Darlington R. B. (2007). The limbic system in mammalian brain evolution. *Brain Behav. Evol.* 70 57–70. 10.1159/000101491 17409735

[B51] Rowe T. B. Macrini T. Luo Z. X. (2011). Fossil evidence on origin of mammalian brain. *Science* 332 955–957. 10.1126/science.1203117 21596988

[B52] Sanai N. Nguyen T. Ihrie R. A. Mirzadeh Z. Tsai H.-H. Wong M. (2011). Corridors of migrating neurons in the human brain and their decline during infancy. *Nature* 478 382–386. 10.1038/nature10487 21964341 PMC3197903

[B53] Shook B. A. Manz D. H. Peters J. J. Kang S. Conover J. C. (2012). Spatiotemporal changes to the subventricular zone stem cell pool through aging. *J. Neurosci.* 32 6947–6956. 10.1523/JNEUROSCI.5987-11.2012 22593063 PMC3359841

[B54] Simic G. Kostovic I. Winblad B. Bogdanovic N. (1997). Volume and number of neurons of the human hippocampal formation in normal aging and Alzheimer’s disease. *J. Comp. Neurol.* 379 482–494. 10.1002/(SICI)1096-9861(19970324)379:4<482::AID-CNE2>3.0.CO;2-Z 9067838

[B55] Sorrells S. F. Paredes M. F. Cebrian-Silla A. Sandoval K. Qi D. Kelley K. W. (2018). Human hippocampal neurogenesis drops sharply in children to undetectable levels in adults. *Nature* 555 377–381. 10.1038/nature25975 29513649 PMC6179355

[B56] Spalding K. L. Bergmann O. Alkass K. Bernard S. Salehpour M. Huttner H. B. (2013). Dynamics of hippocampal neurogenesis in adult humans. *Cell* 153 1219–1227. 10.1016/j.cell.2013.05.002 23746839 PMC4394608

[B57] Suhonen J. O. Peterson D. A. Ray J. Gage F. H. (1996). Differentiation of adult hippocampus-derived progenitors into olfactory neurons in vivo. *Nature* 383 624–627. 10.1038/383624a0 8857538

[B58] Swanson M. E. V. Murray H. C. Oliver M. H. Waldvogel H. J. Firth E. C. Curtis M. A. (2017). Imposed running exercise does not alter cell proliferation in the neurogenic niches of young lambs. *J. Anim. Sci.* 95 4381–4390. 10.2527/jas2017.1710 29108047

[B59] Thewissen J. G. Williams E. M. Roe L. J. Hussain S. T. (2001). Skeletons of terrestrial cetaceans and the relationship of whales to artiodactyls. *Nature* 413 277–281. 10.1038/35095005 11565023

[B60] Tramontin A. D. Garcìa-Verdugo J. M. Lim D. A. Alvarez-Buylla A. (2003). Postnatal development of radial glia and the ventricular zone (VZ): a continuum of the neural stem cell compartment. *Cereb. Cortex* 13 580–587. 10.1093/cercor/13.6.580 12764031

[B61] van Dijk R. M. Huang S. H. Slomianka L. Amrein I. (2016). Taxonomic separation of hippocampal networks: principal cell populations and adult neurogenesis. *Front. Neuroanat.* 10:22. 10.3389/fnana.2016.00022 27013984 PMC4783399

[B62] van Kann E. Cozzi B. Hof P. R. Oelschläger H. H. A. (2017). Qualitative and quantitative analysis of primary neocortical areas in selected mammals. *Brain Behav. Evol.* 90 193–210. 10.1159/000477431 28768268

[B63] Vivar C. van Praag H. (2017). Running changes the brain: the long and the short of it. *Physiology (Bethesda)* 32 410–424. 10.1152/physiol.00017.2017 29021361 PMC6148340

[B64] Wang C. Liu F. Liu Y.-Y. Zhao C.-H. You Y. Wang L. (2011). Identification and characterization of neuroblasts in the subventricular zone and rostral migratory stream of the adult human brain. *Cell Res.* 21 1534–1550. 10.1038/cr.2011.83 21577236 PMC3365638

[B65] Weil Z. M. Norman G. J. De Vries A. C. Nelson R. J. (2008). The injured nervous system: a Darwinian perspective. *Prog. Neurobiol.* 86 48–59. 10.1016/j.pneurobio.2008.06.001 18602443 PMC2662998

[B66] Workman A. D. Charvet C. J. Clancy B. Darlington R. B. Finlay B. L. (2013). Modeling transformations of neurodevelopmental sequences across mammalian species. *J. Neurosci.* 33 7368–7383. 10.1523/JNEUROSCI.5746-12.2013 23616543 PMC3928428

[B67] Zhuo J. M. Tseng H. A. Desai M. Bucklin M. E. Mohammed A. I. Robinson N. T. (2016). Young adult born neurons enhance hippocampal dependent performance via influences on bilateral networks. *Elife* 5:e22429. 10.7554/eLife.22429 27914197 PMC5156524

